# Photosynthetic recovery and acclimation to excess light intensity in the rehydrated lichen soil crusts

**DOI:** 10.1371/journal.pone.0172537

**Published:** 2017-03-03

**Authors:** Li Wu, Yaping Lei, Shubin Lan, Chunxiang Hu

**Affiliations:** 1 School of Resources and Environmental Engineering, Wuhan University of Technology, Wuhan, China; 2 Key Laboratory of Algal Biology, Institute of Hydrobiology, Chinese Academy of Sciences, Wuhan, China; Institute for Sustainable Plant Protection, C.N.R., ITALY

## Abstract

As an important successional stage and main type of biological soil crusts (BSCs) in Shapotou region of China (southeastern edge of Tengger Desert), lichen soil crusts (LSCs) often suffer from many stresses, such as desiccation and excess light intensity. In this study, the chlorophyll fluorescence and CO_2_ exchange in the rehydrated LSCs were detected under a series of photosynthetically active radiation (PAR) gradients to study the photosynthetic acclimation of LSCs. The results showed that although desiccation leaded to the loss of photosynthetic activity in LSCs, the fluorescence parameters including Fo, Fv and Fv/Fm of LSCs could be well recovered after rehydration. After the recovery of photosynthetic activity, the effective photosynthetic efficiency Φ_PSII_ detected by Imaging PAM had declined to nearly 0 within both the lichen thallus upper and lower layers when the PAR increased to 200 μE m^-2^ s^-1^, however the net photosynthesis detected by the CO_2_ gas analyzer in the LSCs still appeared when the PAR increased to 1000 μE m^-2^ s^-1^. Our results indicate that LSCs acclimating to high PAR, on the one hand is ascribed to the special structure in crust lichens, making the incident light into the lichen thallus be weakened; on the other hand the massive accumulation of photosynthetic pigments in LSCs also provides a protective barrier for the photosynthetic organisms against radiation damage. Furthermore, the excessive light energy absorbed by crust lichens is also possibly dissipated by the increasing non-photochemical quenching, therefore to some extent providing some protection for LSCs.

## Introduction

In desert regions, most of vegetation is limited due to the extreme environmental stresses such as drought, UV radiation, salinity, wind and sand-scouring. However biological soil crusts (BSCs) can survive those stresses and develop in these regions, because of their unique physiological and ecological characteristics, and even accounting for up to 70% of the living cover in some areas [1, 2]. Having an important ecological role in desert regions, BSCs are a complex mosaic of soil particles and organisms within the uppermost millimeters of soil surface, in which cyanobacteria (especially the filamentous ones) first settle and grow, then other organism such as lichens and mosses emerge after the topsoil is stabilized [3–5]. Subsequently, BSCs gradually develop to the stages of lichen soil crusts (LSCs), in which lichens are dominant although many other organisms, such as cyanobacteria, algae, and mosses, also co-exist in the crusts [6, 7].

In desert regions, BSCs are in dry state during most of the time because of the severely limited precipitation, and the dehydrated BSCs have no metabolic activity. It has been demonstrated that desiccation could lead to the inactivity of light-harvesting complexes (LHCs) and reaction centres of photosystem (PS) II in crust photosynthetic organisms [8, 9], the functional disconnection of light-harvesting complexs II (LHCs II) from reaction centres [10], and also the release of manganese clusters and the conformational changes of chlorophyll-proteins in or near PS II centers to dissipate the excess energy [11, 12]. However, although no photosynthetic activity was detected in BSCs when they are in dry state, once water is gained, Harel et al. [13] reported cyanobacterial soil crusts would rapidly recover their photosynthetic activity, and energy transfer to PS II and PS I by the respective antennae was fully established within 10 to 20 min of rehydration. Similarly the mechanism of photosynthetic protection against desiccation has also been well documented in lichens [14–18]. After rehydration, the dry lichen photosynthetic apparatus could reconstitute and their photosynthetic activity could recover rapidly [19–21].

Although lichens are dominant in LSCs, other photosynthetic organisms also exist, and additionally some soil microbes would further affect crust CO_2_ exchange and net photosynthetic efficiency [7, 22]. Therefore, studying the photosynthetic patterns of the rehydrated LSCs would help us further understand the photosynthetic performance of this type of BSCs in their natural environments. After rehydration, the photosynthetic organisms directly distributed on LSC surface receive light energy more easily to start photosynthesis, which in return make LSCs often expose to the excess light intensity. Therefore, in the present study we monitored the photosynthetic patterns of the rehydrated LSCs under different light intensity conditions using chlorophyll fluorescence and gas exchange techniques, in order to understand the photosynthetic performance and explore the photosynthetic acclimation in the rehydrated LSCs. The results will not only help us understand the development, succession and ecological acclimation of BSCs in desert environmental conditions, but also provide some useful information for the maintenance and management of BSCs in desertification control.

## Materials and methods

### Sampling

The LSC samples used in this experiment were collected from a non-irrigated revegetation region at the Shapotou Desert Research and Experimentation Station of the Chinese Academy of Sciences (located at the southeastern edge of Tengger Desert; 37°27′N, 104°57′E). This region was a steppe-desert transition zone, widely covered with BSCs (mostly LSCs) and more details about this experimental region were described in the previous studies [7, 22]. In our experiment, the LSC samples dominated by *Collema tenax* were collected to study their photosynthetic acclimation to the excess light intensity. In the collected LSCs, the dominant lichens occupied most of the crust biomass and more than 60% of the crust surface during the dry period, and even 100% of the crust surface when the crusts were moistened [6, 7]. In addition, some cyanobacteria soil crusts were also sampled, in which *Microcoleus vaginatus* and *Scytonema javanicum* were the dominant species, and the abundance of *M*. *vaginatus* was more than 80%. Before sampling, the experimental region had not received any rainfall in the latest one week, and the water content of all the collected crust samples was less than 0.6% (Table 1). After collection, the crust samples were transported back to the laboratory as quickly as possible and stored in desiccators (relative humidity about 20%) for the subsequent analysis.

**Table 1:** Basic chareacteristics of BSCs in the experiment.

	Cyanobacterial soil crusts	Lichen soil crusts
Color	Gray	Dark
Surface	Flat	Coarse
Thickness (mm)	3–5	6–8
Dominant species	*Microcoleus vaginatus*; *Scytonema javanicum*	*Collema tenax* (cyanolichen)
Water content (%)	<0.5	<0.6
Cyanobacteira coverage (%)	>95	<20
Lichen coverage (%)	0	>70
Moss coverage (%)	<5	<10
Sand percentage (%)	86.9 ± 2.6	64.0 ± 4.7
Silt percentage (%)	12.6 ± 2.4	34.5 ± 4.4
Clay percentage (%)	0.5 ± 0.2	1.5 ± 0.4

### The recovery of LSCs after rehydration

In order to recover the photosynthetic activity, three LSC samples were fully rehydrated with sterilized distilled water. The rehydrated LSCs were then transported to a green house (25 ± 1°C) and recovered in the light at 40 μE m^-2^ s^-1^ [9]. During the experiment, LSCs were weighed and hydrated every hour to ensure the constancy of water conditions. Chlorophyll (Chl) fluorescence parameters of the rehydrated LSCs were measured at an intensive interval period using a Plant Efficiency Analyzer (Handy PEA, Hansatech Instruments Ltd., UK). Before each measure, the LSC samples were dark-adapted at least 10 min, then a saturating pulse of approximately 3000 μE m^-2^ s^-1^ was supplied to excite the samples by the Handy PEA. The fluorescence transients were automatically recorded by the Handy PEA for 2 s. The fluorescence intensity at 50 μs, when the quinone Q_A_ was fully oxidized and all of the PS II centers were open, was designated as original fluorescence (Fo); the fluorescence at 2 ms was designated the J step, and at 30 ms the I step [23, 24]. The maximal fluorescence gained in the fluorescence transients was Fm, then the variable fluorescence (Fv) was calculated by Fm—Fo, and the Fv/Fm ratio, the maximal quantum yield of PS II [25], was also calculated (Table 2).

**Table 2:** Chlorophyll fluorescence parameters used in the experiment and relative physiological significance.

Parameter	Definition	Physiological significance
Fo	Original (minimal) fluorescence	Fluorescence intensity when QA is maximally oxidized (PS II centers open)
Fv	Variable fluorescence	Related to the photochemical efficiency of PS II, an indicator of charge separation of PS II centers
Fv/Fm	Maximum quantum yield of PS II photochemistry	Maximum quantum yield at which light absorbed by PS II is used for reduction of QA, an indicator of the photosynthetic activity of PS II
Φ_PSII_	Effective quantum yield of PS II photochemistry	Quantum yield of PS II at a given light condition, reflecting the light energy that is absorbed by PS II and used in photochemistry
qP	Photochemical quenching	Fluorescence yield is lowered because of use of excitation energy for photochemical reactions
qN	Non-photochemical quenching	Fluorescence yield is lowered because of energy dissipation in the form of heat
rETR	Relative electron transport rate	Performance of electron transport chain in PS II

### The effects of light intensity on Chl fluorescence parameters

After fully photosynthetic recovery (Fv/Fm maintained at a constant level) in the rehydrated LSCs, lichen thalli separated from the surface of LSC samples were vertically cut into slices, and these vertical sections of lichen thalli were observed under a Microscope-Imaging PAM (Walz, Germany). The Chl fluorescence information from thallus surface to substrate was determined and given as the false color images to exhibit the relative values of each fluorescence parameter, in which different colors indicated the different values of each parameter, and the maximum value of each parameter was adjusted to 1; white and black represented 1 and 0, respectively. During each measure, 8 light intensities (0, 17, 26, 54, 81, 108, 154 and 200 μE m^-2^ s^-1^) were set with a pre-installed software routine, the light was provided from side direction to the cross section, and the illumination duration of each light intensity was 10 s. Then the fluorescence parameters including effective fluorescence parameters Φ_PSII_, photochemical quenching qP, non-photochemical quenching qN and relative electron transfer rate rETR (Table 2) under a series of photosynthetically active radiation (PAR) gradients were automatically determined and recorded by the pre-installed software routine.

### The effects of light intensity on CO_2_ exchange

According to the described methods of Wu et al. [22], another set of fully recovered LSCs were placed in a closed incubator, illuminated at a series of PAR gradients from 0–1000 μE m^-2^ s^-1^. Under each PAR gradient, LSCs were rehydrated to constant weight (just saturated) and their CO_2_ exchange rate was analyzed by infrared CO_2_ gas analyzer (S157, Qubit, Canada) for at least 5 min and calculated as follow:

P=C×V×273×1000/(t×(273+T)×22.4×S)

In which, P is the CO_2_ exchange rate (nmol cm^-2^ min^-1^); C is the change of the CO_2_ content in the incubator detected by the infrared gas analyzer during the measure time (ppm); V (0.164L) and T (25°C) are the volume and temperature of the incubator respectively; t is the measuring time (min); S is the area of the crusts (cm^2^); 273 and 22.4 are Kelvin temperature (K) and molar volume of gas (L mol^-1^) under standard temperature and pressure.

### Chl-*a*, carotenoids and scytonemin determination

In order to better comprehend and compare the contents of Chl-*a*, carotenoids and scytonemin in LSCs, some cyanobacteria soil crusts were also studied in this part of experiment. For pigment determination, all the LSC samples and cyanobacterial soil crusts were first ground in acetone, and the extractions were conducted at 4°C in darkness overnight. Then the contents of Chl-*a*, carotenoids and scytonemin were determined spectrometrically with the trichromatic equations of Garcia-Pichel and Castenholz [26] and Garcia-Pichel et al. [27].

### Data analysis

All the experiments were conducted three times as repetitions. The differences of fluorescence parameters Φ_PSII_, qP, qN and rETR between thallus upper and lower layers were analyzed using Paired Samples T-Test, and the differences of Chl-*a*, carotenoids and scytonemin between cyanobacterial soil crusts and LSCs was analyzed using Independent Samples T-Test. The variance of CO_2_ exchange among different PAR was analyzed using One-way ANOVA. All above data analyses were carried out using SPSS13.0 software (SPSS Inc., USA). In addition, in order to evaluate the changing trends of Fv and Fv/Fm after LSCs were rehydrated, curve fitting between Fv or Fv/Fm and recovery time was performed via Curve Estimation (Hyperbola) using SigmaPlot 12.5 software (Systat Software Inc., USA).

## Results

### Recovery of Chl fluorescence in the rehydrated LSCs

**Fig 1:**
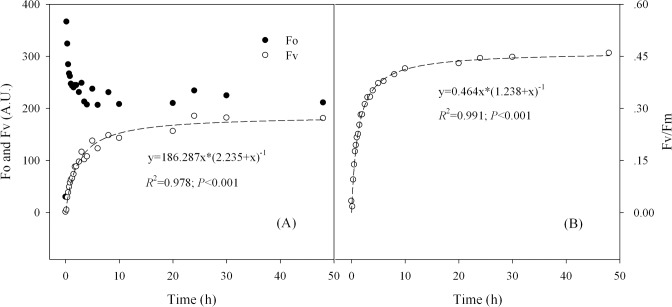
**Fitting curves of the recovered Fo, Fv (A) and Fv/Fm (B) and their corresponding fitting equations in the rehydrated lichen soil crusts.** Each fluorescence parameter just show the results of one sample from the three similar repetitions, and the other repetitive results are given in the Figure A in S1 File.

**Fig 2:**
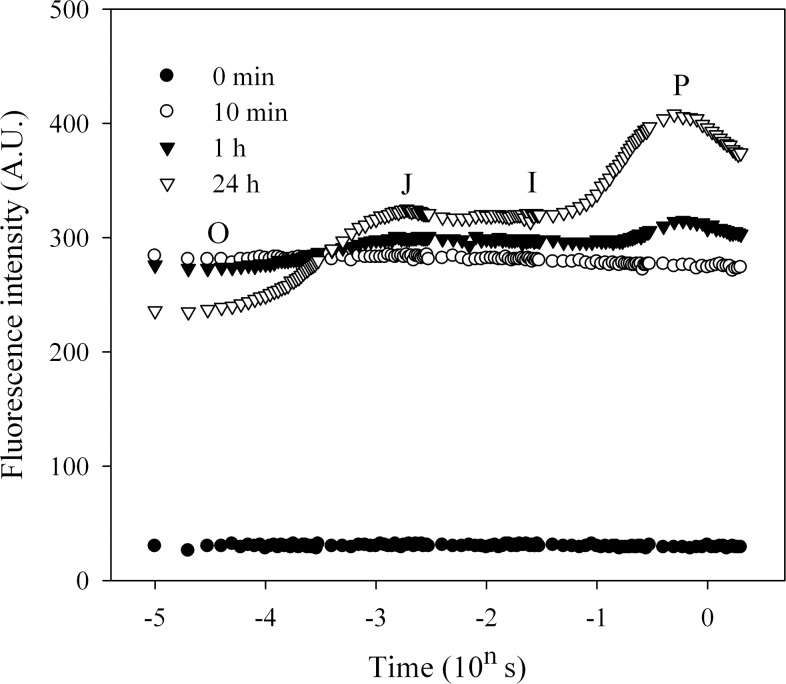
The recovery of chlorophyll fluorescence transients (O-J-I-P kinetic curves) in the rehydrated lichen soil crusts. Each kinetic curve just show the results of one sample from the three similar repetitions, and the other repetitive results are given in the Figure B in S1 File.

When the LSC samples were in dry state, no fluorescence signal could be excited from them. Once the LSCs were rehydrated, their fluorescence signals rapidly recovered, and during the recovery process different fluorescence parameters showed the different recovery patterns (Fig 1 and Figure A in S1 File). After rehydration, Fo reached the maximum value within 10 min, and then decreased to a relative stable level in the following recovery time (Fig 1A). In the rehydration process, the two parameters Fv and Fv/Fm fully changed according to the function f(x) = ax*(b+x)^-1^ (*P*<0.001; Fig 1B). In addition, it was found that the fluorescence transients of dry LSCs were almost a straight horizontal line (Fig 2 and Figure A in S1 File). After rehydration, fluorescence signal increased significantly within the 10 min, however no obvious O-J-I-P steps appeared during this recovery period. Although the P-step was shown after 1 h, Fv was still low at this time; additionally, both the J-step and I-step were indistinguishable before 1 h of recovery. Until 24 h of rehydration, the high Fv, Fv/Fm (reached up to 0.44–0.47), as well as distinctive O-J-I-P steps were not observed although Fo was lower at this time than that at 1 h (or 10 min; Fig 2 and Figure B in S1 File).

### The effects of light intensity on Chl fluorescence parameters

**Fig 3:**
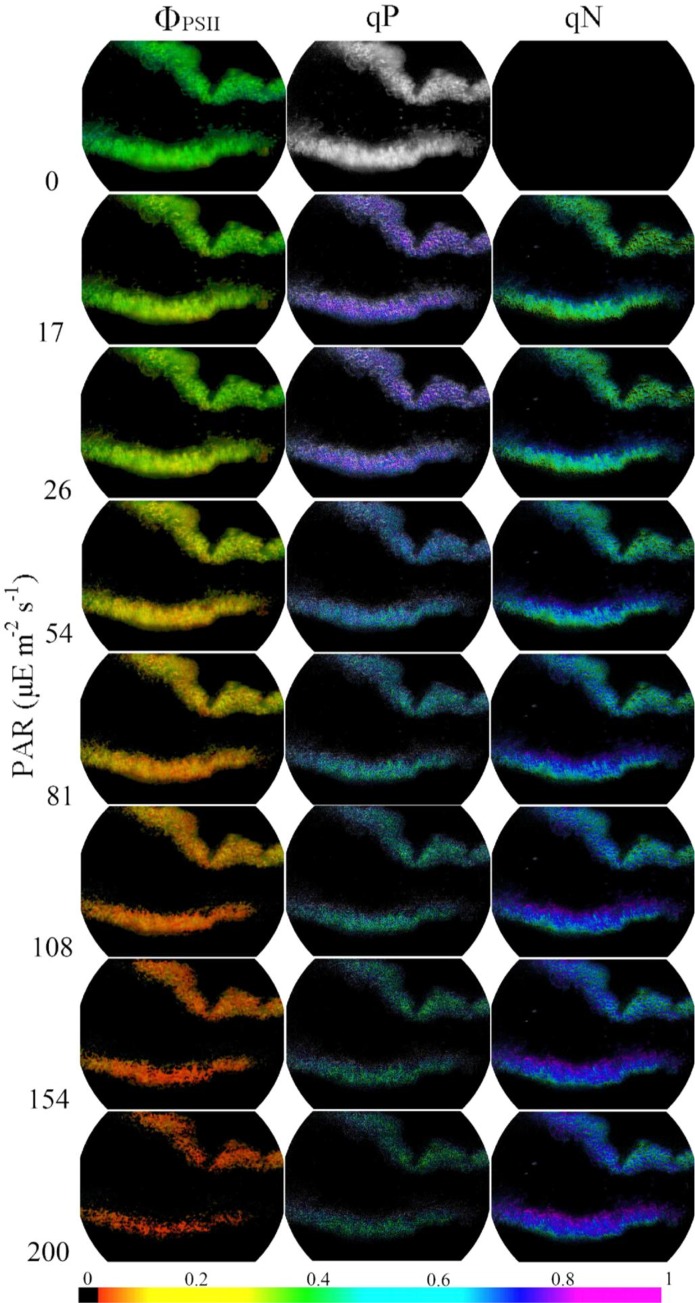
Images of Φ_PSII_, qP and qN measured under different photosynthetically active radiation in the rehydrated lichen thallus. Different colors (bar at the bottom) indicate different values of each parameter, and the maximum value of each parameter has been adjusted to 1. All these pictures are a vertical section of the lichen thallus with its upper cortex up. Each fluorescence parameter just show the results of one sample from the three similar repetitions, and the other repetitive results are given in the Figure C in S1 File.

**Fig 4:**
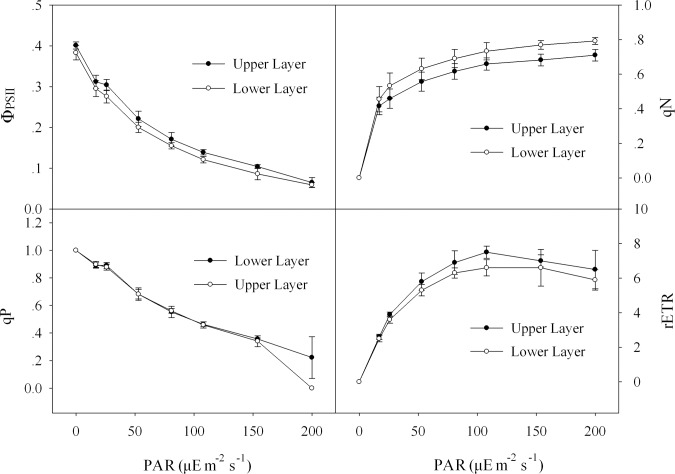
Chlorophyll fluorescence parameters of upper layer and lower layer in the rehydrated lichen thallus. Data indicate means ± SD (*n* = 3).

After the photosynthetic activity of lichen thallus fully recovered, the Chl fluorescence parameters including Φ_PSII_, qP and qN under different PAR were displayed in the form of false color images, with red to purple representing 0–1 (black and grey scale means 0 and 1 respectively; Fig 3 and Figure C in S1 File). From the color images it was found with the increase of PAR, the colors of Φ_PSII_ and qP changed from green to orange-red and indigo-blue (leave the grey image out of account) to blue-green respectively, which indicated the decrease of parameters Φ_PSII_ and qP. While with the increase of PAR, the parameter qN gradually increased, reflected by the color changing from green (similarly leave the black image out of account) to indigo-blue. The increase of qN, decrease of ΦPSII and qP with PAR also could be presented in detail in values as showed in Fig 4. In addition, with the increase of PAR, the rETR in lichen thallus first increased and then followed by a slight decrease. From the vertical gradient, no obvious difference was found in any fluorescence parameters (including Φ_PSII_, qP, qN and rETR) between the thallus upper and lower layers (*P*>0.05; Fig 4).

### The effects of light intensity on CO_2_ exchange

**Fig 5:**
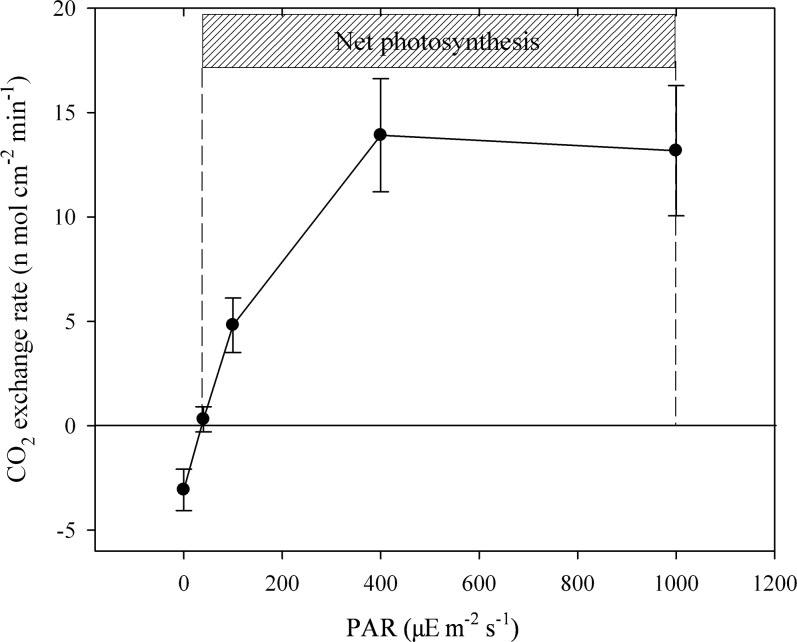
CO_2_ exchange rate of the rehydrated LSCs under different photosynthetically active radiation. Data indicate means ±SD (*n* = 3).

No CO_2_ exchange was detected in the dry LSCs neither in the light nor the dark. After the LSCs were fully rehydrated, the CO_2_ exchange rate gradually increased with the increase of PAR from darkness. Then the CO_2_ exchange rate reached a plateau to steady state when the PAR continued to increase from 400 to 1000 μE m^-2^ s^-1^ (*P*>0.05; Fig 5). In addition, it was obviously found that CO_2_ assimilation had occurred when the PAR was 40 μE m^-2^ s^-1^ (light compensation point), and even the PAR increased to 1000 μE m^-2^ s^-1^, CO_2_ assimilation could still be detected (Fig 5).

### The contents of Chl-*a*, carotenoids and scytonemin in LSCs

**Fig 6:**
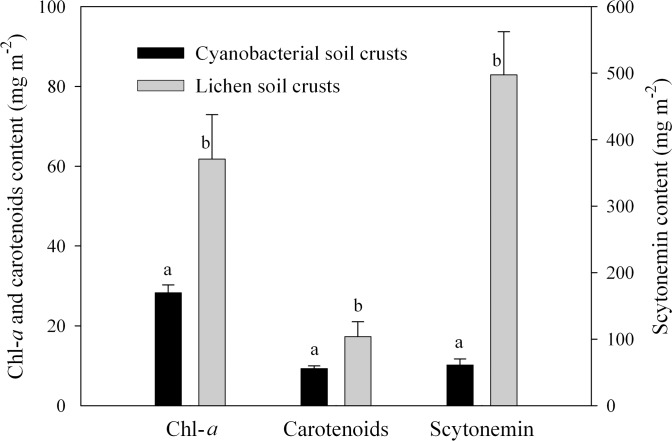
The contents of Chl-*a*, carotenoids and scytonemin in cyanobacterial soil crusts and lichen soil crusts. Data indicate means ±SD (*n* = 3).

Three types of pigments, including Chl-*a*, carotenoids and scytonemin, appeared in both cyanobacterial soil crusts and LSCs, and all these pigments were higher in LSCs than those in cyanobacterial soil crusts (Fig 6). The Chl-*a* and carotenoids content of LSCs were about 2.18 times and 1.86 times higher than those of cyanobacterial soil crusts, respectively (*P*<0.05), while the scytonemin content of LSCs was head and shoulders above that of cyanobacterial soil crusts (8.14 times, *P*<0.05). As a result, no significant difference in the ratio of carotenoids to Chl-*a* was found between the two types of BSCs (*P*>0.05), while the ratio of scytonemin to Chl-*a* was much higher in LSCs (*P*<0.05).

## Discussion

As the important successional stage of BSCs, LSCs always develop and succeed from cyanobacterial soil crusts, and both of them in desert regions would experience the particular stress conditions, such as desiccation and excess light intensity. Different from the dominant cyanobacteria dwelling in the subsurface of cyanobacterial soil crusts, lichens directly distributed on the surface of LSCs, therefore LSCs have the extraordinary advantage in water and light competition. However, on the other hand, the distribution position also exposes LSCs to the harsher extreme environments.

Chl fluorescence provides a subtle indication on the photochemistry of photosynthesis and has been wildly used in ecophysiological studies [25, 28, 29]. Under unstressed conditions, Fv/Fm, as an indicator of the photosynthetic activity of photosystem II (PS II), remains at a relative stable level and decreases when the photosynthetic organisms are under stressed conditions [8, 22]. The variable fluorescence Fv, obtained by subtracting original fluorescence Fo from maximal fluorescence Fm, is a good indicator of charge separation of PSII centers [18]. When LSCs were in the dry state, no photosyhtnetic activity was detected, which was indicated by the low Fv and Fv/Fm. After rehydration, the original fluorescence Fo first reached the maximum, indicating light harvesting complexes (LHCs) could rapidly recover the function, and the following drop of Fo to a relatively stable state might imply us that the energy transfer from LHCs to reaction center was gradually established. During that course, the photosynthetic activity of PS II centers was gradually recovered, indicated by the increasing Fv and Fv/Fm. The recovery of Fo was faster than that of Fv or Fv/Fm, might imply us the photosynthetic recovery in PS II centers was (or at least partly) light energy induced or dependent.

After the recovery of photosynthetic activity, the photosynthesis of LSCs was also affected by the light intensity conditions. In our previous study [9], it was found the effective photosynthetic efficiency, indicated by chlorophyll fluorescence parameter Φ_PSII_, in both cyanobacterial and moss soil crusts decreased with the increase of PAR from morning to noon. Similarly in the present study, it was also found Φ_PSII_ and photochemical quenching qP in lichen thallus decreased with the increasing PAR, reflecting the decrease of photosynthetic efficiency in LSCs. When the photochemical efficiency decreased, the excessive excitation energy was possibly dissipated by the increasing non-photochemical quenching qN under the high PAR. With the increase of PAR, the relative electron transfer rate (rETR) in lichen thallus first increased and then followed by a slight decrease, reflecting the electron transporting efficiency of photosynthetic systems was down-regulated when PAR exceeded 108 μE mol m^-2^ s^-1^. In addition, no significant difference in Ф_PSII_, qP and rETR was found between lichen thallus upper and lower layers, which implied us the excess light intensity in desert regions (generally about 2000 μE m^-2^ s^-1^ at summer noon), did not significantly affect the photosynthetic activity of photobiont in lichens, at least the effect was reversible or repairable. This further demonstrated the protection in LSCs providing for photosynthetic organisms was relatively complete.

In our experiments, when the PAR increased to 200 μE m^-2^ s^-1^, the photosynthetic efficiency indicated by Chl fluorescence parameter ФPSII in both lichen thallus upper and lower layers had declined to nearly 0. To the contrary, the results of CO_2_ gas analyzer showed that net photosynthesis still appeared even when the PAR increased to 1000 μE m^-2^ s^-1^. In addition, Lange et al. [19] reported that *Collema tenax* isolated from LSCs had very high light saturating points of 2000 μE m^-2^ s^-1^ at 30°C and 1600 μE m^-2^ s^-1^ at 26°C. Those seeming photosynthetic differences among the different monitoring experiments are mainly due to the following three reasons: 1) the variable fluorescence Fv is uncoupling with photosynthetic O_2_ evolution or CO_2_ gas fixation in crust photosynthetic organisms, the back electron flow and charge recombination may be pheophytin-independent and mainly via a non-radiative route [22, 30, 31]; 2) the different biological communities affect photosynthetic performance. For example, in our present study, the Chl fluorescence was excitated from the limited depth of LSCs, but CO_2_ exchange was the comprehensive reflection of photosynthesis and respiration in the whole LSCs; whereas in the report of Lange et al. [19], the CO_2_ exchange just came from crust lichen *Collema tenax*; 3) the different environmental conditions also affect photosynthetic performance. In our study the LSCs were fully rehydrated, while not in the experiment of high light saturating points of Lange et al. [19], because they found light saturation of *Collema tenax* was strongly dependent on hydration condition, and the photosynthetic CO_2_ fixation of *Collema tenax* was already light saturation at lower PAR when the lichen was fully rehydrated [19].

For determining Chl fluorescence parameter under an Imaging PAM, the incident light was provided by the Imaging PAM. In that case, the lower layer of lichen thallus could be directly illuminated, since the vertical sections of crust lichens were observed. However, in the actual case of field conditions, even if the environmental light intensity is high enough, the incident light to the lower layer of lichen thallus can be still very limited, which ensures the inner photosynthetic organisms in LSCs to be under a more appropriate light intensity. Thus, in our experiments it was also found that net photosynthesis still occurred even when the LSCs were at a high light intensity up to 1000 μE m^-2^ s^-1^. In arid and semiarid desert regions, although PAR often excels 1000 μE m^-2^ s^-1^ at noon, LSCs generally are dehydrated at that time. Under that situation, most light energy is dissipated in the form of heat through non-photochemical quenching [29]. When LSCs start photosynthesis after obtaining water from dewfall (early morning) or precipitation (cloudy day), the PAR generally is low. In addition, LSCs can reduce radiation damage through the accumulation of protective pigments, such as carotenoids and scytonemin. As anultraviolet absorbing pigment, scytonemin can effectively provide ultraviolet radiation protection to photosynthetic organisms [26, 27,32]; while carotenoids is considered as an important photosynthetic protective pigment against oxidative damage [8, 31].

In our experimental region, with high PAR as common environmental condition, most BSCs developed and succeeded to LSCs, which covered more than 50% of the soil surface [7, 22]. That may also reflect the excellent photosynthetic acclimation to excess light intensity in LSCs. In the field, most of time LSCs are dehydrated, and once the dry LSCs are rehydrated, they would well recover their photosynthetic activity, and conduct photosynthesis, although the photosynthetic efficiency may decrease with the increasing PAR. The fact that LSCs could acclimate to high PAR, can be considered as the result of the special structure and photosynthetic charateristics in LSCs, making the incident light into lichen thallus be weakened; the excessive light energy be absorbed by protective pigments or dissipated by increasing non-photochemical quenching.

## Supporting information

S1 File Supplementary Figures A to C.
(DOC)
