# Supplementary material for: Photosynthetic recovery and acclimation to excess light intensity in the rehydrated lichen soil crusts
Source: PLoS One. 2017 Mar 3;12(3):e0172537. doi: 10.1371/journal.pone.0172537 (PMC5336202; doi:10.1371/journal.pone.0172537)
Supplement: S1 File — Supplementary Figures A to C. (DOC) [file pone.0172537.s001.doc]

**Supporting information: S1 File**

**Full title:** Photosynthetic recovery and acclimation to excess light intensity in the rehydrated lichen soil crusts

**Authors:** Li Wu ^1^, Yaping Lei ^1^, Shubin Lan ^2*^, Chunxiang Hu ^2^

^1^ School of Resources and Environmental Engineering, Wuhan University of Technology, Wuhan, 430072, China

^2^ Key Laboratory of Algal Biology, Institute of Hydrobiology, Chinese Academy of Sciences, Wuhan, 430072, China

* Corresponding author: Key Laboratory of Algal Biology, Institute of Hydrobiology, Chinese Academy of Sciences, Wuhan, 430072, China. Tel/Fax.: +86 27 68780046; E-mail address: shblan@ihb.ac.cn (S.B. Lan)

**Number of supplementary figs:** 3

**Fig A.** Fitting curves of the recovered Fo, Fv and Fv/Fm and their corresponding fitting equations in the rehydrated lichen soil crusts. The top and bottom figures show the results of the two similar repetitions, respectively.

**Fig B.** The recovery of chlorophyll fluorescence transients (O-J-I-P kinetic curves) in the rehydrated lichen soil crusts. The left and right figures show the results of the two similar repetitions, respectively.


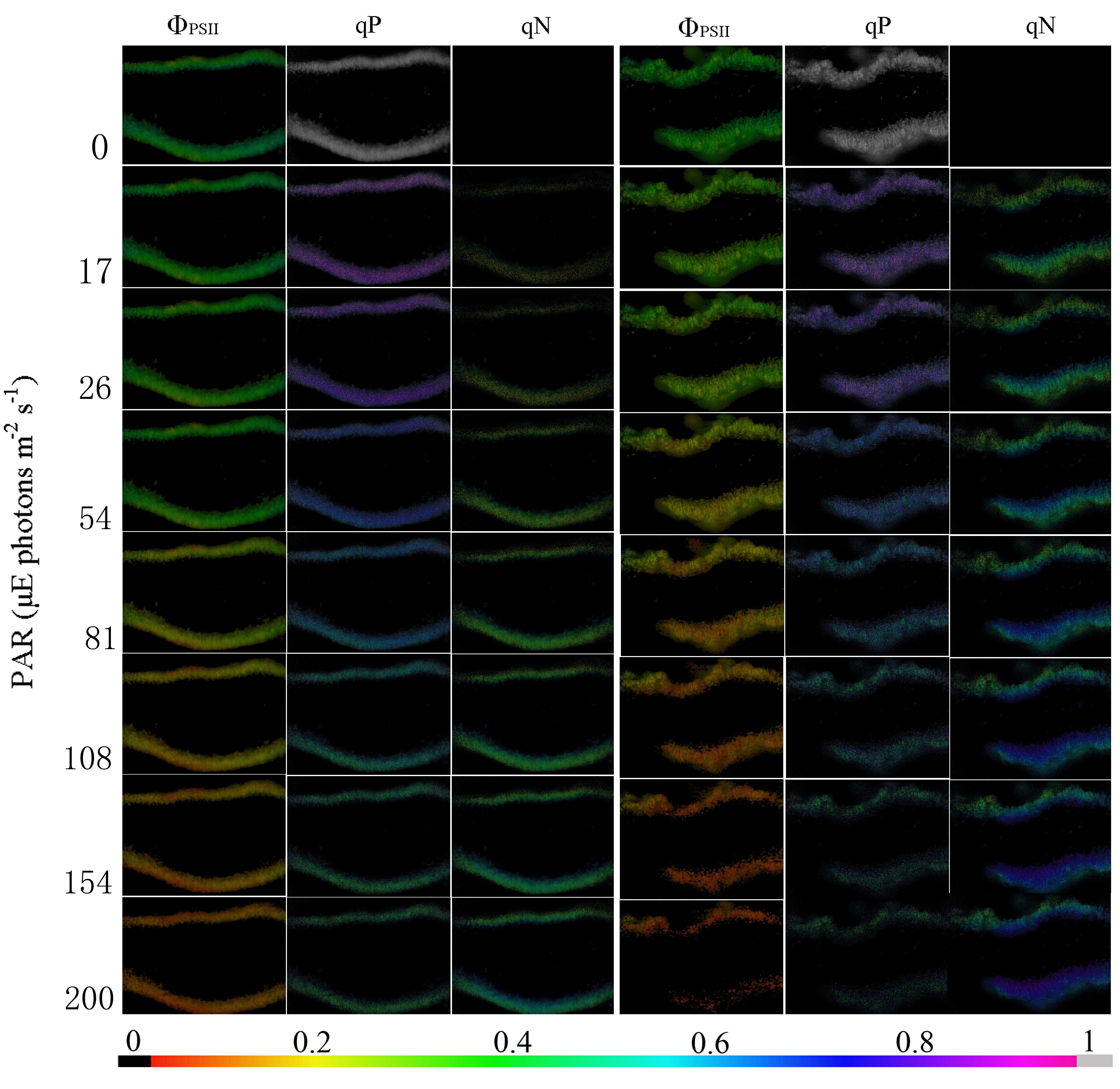


**Fig C.** Images of Φ_PSII_, qP and qN measured under different photosynthetically active radiation in the rehydrated lichen thallus. Different colors (bar at the bottom) indicate different values of each parameter, and the maximum value of each parameter has been adjusted to 1. All these pictures are a vertical section of the lichen thallus with its upper cortex up. The left and right figures show the results of the two similar repetitions, respectively.
